# A Study to Improve the Reliability of High-Strength Concrete Strength Evaluation Using an Ultrasonic Velocity Method

**DOI:** 10.3390/ma16206800

**Published:** 2023-10-21

**Authors:** Wonchang Kim, Taegyu Lee

**Affiliations:** Department of Fire and Disaster Prevention, Semyung University, Jecheon 27136, Republic of Korea; kimwc69082@gmail.com

**Keywords:** concrete, ultrasonic pulse velocity, high temperature, prediction model, statistical analysis

## Abstract

The ultrasonic pulse velocity (UPV) technique, which is an efficient technique for concrete quality evaluation, can be affected by several factors. Many studies have proposed compressive-strength prediction models based on UPV in concrete; however, few studies have investigated the factors resulting in statistically different UPV results for different models. This study examined the difference between compressive strengths of various concrete specimens calculated by age-dependent and temperature-dependent UPV-based prediction models. Furthermore, a statistical analysis was conducted to evaluate the influence of aggregates and water/cement ratio (design compressive strength), which are said to affect UPV, on the compressive-strength prediction models. The experimental results revealed that the residual compressive strength of concrete after high-temperature exposure was about 9.5 to 24.8% higher than the age-dependent compressive strength. By contrast, after high-temperature exposure, UPV tended to be about 34.5% lower. The compressive strengths and UPVs were significantly different with respect to high temperature, aggregate density, and design compressive strength. The compressive-strength prediction model derived from the regression analysis showed a high R^2^ (average 0.91) and mean error converged to zero compared to the compressive-strength prediction model without considering these factors. Finally, the differences between the age- and temperature-based compressive-strength prediction models were analyzed according to the corresponding microstructures.

## 1. Introduction

Sound waves whose frequencies are beyond the audible range are called ultrasound. The transmission of sound through a medium is driven by the kinetic energy of vibrating particles in the medium and the resulting motion of the surrounding particles [1]. The transmission time and distance traveled by sound waves in a material can be used to measure the velocity of the waves in the material and evaluate the structural quality of the material. Initially, ultrasound waves were used to evaluate defects in man-made steel constructions such as bridges, steel structures, and piping [2,3]. Steel is a physically undifferentiated homogeneous material, which is artificially manufactured in factories through processes such as cutting, pressing, and grinding. Hence, only a few factors affect the outcomes of ultrasonic flaw detection in steel. However, concrete, composed of various inorganic materials, is a relatively inhomogeneous material. Even with the same raw materials, the concrete matrix structure can vary depending on the mix proportions, curing conditions, temperature, load, and vibration of concrete. This can affect the results of concrete flaw detection using the ultrasonic pulse velocity (UPV) technique and impede the establishment of generalized quality evaluation criteria and indicators [4,5]. Therefore, the various factors affecting the detection of concrete flaws must be continuously investigated and analyzed to accurately evaluate concrete quality using UPV.

**Figure 1:**
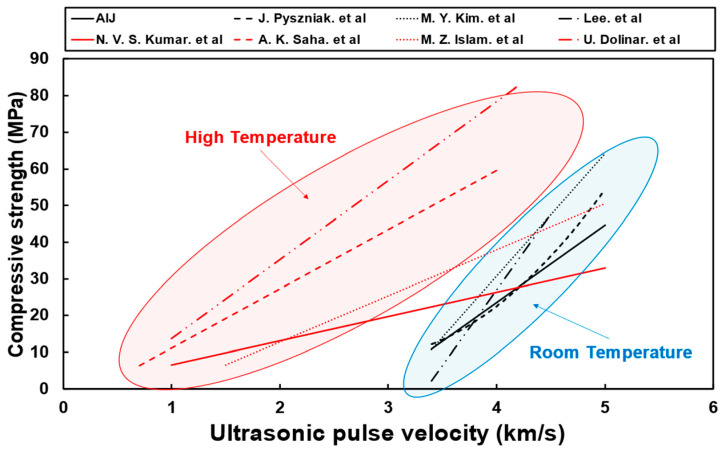
Proposed prediction model of compressive strength on concrete [6,7,8,9,10,11,12,13].

Previous studies conducted on the prediction of concrete strength using UPV primarily employed two approaches: the analyses of (1) compressive strength and UPV with respect to concrete age and (2) residual strength and UPV after exposing the concrete to high temperatures. Figure 1 shows the results of previously reported concrete compressive-strength prediction models [6,7,8,9,10,11,12,13]. The black linear model below is the strength prediction model for concrete that has not been subjected to high temperature (room temperature), and the red linear model above is the strength prediction model for concrete that has been subjected to high temperature. Overall, at room temperature, the models exhibited a UPV of approximately 3 km/s and 5 km/s at an age of 1 day and 91 days, respectively. However, for concrete exposed to high temperatures, the models exhibited a UPV of approximately 1.5 km/s. This is attributed to the relationship between the temperature-dependent matrix structure of concrete and UPV. Most of the recent research trends through UPV analysis are predictions of strength through computer programming such as machine learning and deep learning for elements such as materials and mixing and existing prediction formulas. However, it is also considered important to analyze and evaluate the differences between UPV and predictive models caused by material factors [14,15,16].

**Figure 2:**
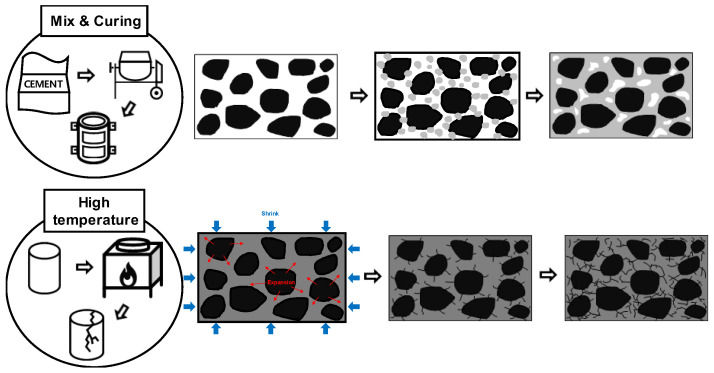
Conception of cement matrix during curing and after high temperature.

Conceptual diagrams of the concrete matrix during curing and after high-temperature exposure are shown in Figure 2. Concrete is converted from a liquid to a solid phase through hydration immediately after mixing. After an age of 1 day, the UPV is predominantly influenced by the elasticity, stresses at the aggregate–paste interface, and voids, depending on the amount of hydration products. Moreover, the UPV is also partially influenced by the cracks formed due to the physical shrinkage of concrete during hydration. The speed of sound is given by the following expression:

(1) $$c=\sqrt{\frac{K}{\rho }},$$

where c, K, and ρ are the sound velocity, elastic modulus, and density, respectively.

As evident from Equation (1), UPV is proportional to the square root of the elastic modulus, which is primarily influenced by the number of coarse aggregates and the water/cement (W/C) ratio. Aggregates account for approximately 70% of concrete by volume, and the W/C ratio affects the compressive strength.

However, UPV in concrete after high-temperature exposure exhibits a different trend. Concrete exhibits higher strength at high temperatures than at room temperature for the same UPV range (see Figure 1). This is because the strength resulting from the hydration reactions converges to a certain extent at high temperatures. Nevertheless, when subjected to high temperatures, cracks develop at the interface due to aggregate expansion and paste shrinkage, and voids and cracks develop in the matrix due to dehydration and cement decomposition (Table 1) [17,18,19,20,21,22]. Hence, UPV reduces significantly (see Figure 2). UPV is also strongly influenced by the thermal expansion characteristics of the different types of concrete aggregates and the amount of cement per unit volume of concrete, as expressed by the W/C ratio [23,24]. Consequently, researchers and engineers must understand the relationship between concrete and UPV to study and establish accurate compressive-strength prediction models of concrete, the utilization of which is crucial for the evaluation of concrete quality.

Several researchers have proposed compressive-strength prediction models for concrete. However, few studies have investigated the differences between compressive-strength prediction models based on the analysis of age-specific and residual mechanical properties after high-temperature exposure. Moreover, the validation of these models remains sparsely documented. Additionally, for an accurate compressive-strength prediction model for concrete, it is essential to determine the distribution pattern of the data and select a regression model that can be effectively fitted to this distribution. In addition, a wide distribution of data may entail data classification prior to the selection of a suitable model. The majority of previous studies either classified the data from a material-centric perspective or developed compressive-strength prediction models based on the entire dataset. Few studies have analyzed the factors leading to different results obtained by different compressive-strength prediction models.

This study evaluated the differences between the concrete compressive-strength prediction models based on age and high-temperature exposure. Subsequently, we employed hypothesis testing, a statistical analysis technique, to evaluate the significant differences between age-specific and temperature-exposure-based compressive strengths and UPVs. The null hypothesis stated that there is no statistically significant difference in concrete compressive strength and UPV depending on the set factors (high-temperature history, aggregate density, and design compressive strength). The alternate hypothesis stated that there is a significant difference depending on each set factor.

Finally, in this study, an analysis was conducted to propose a model for accurate prediction of compressive strength of concrete according to the high temperature using UPV. In addition, an error test was performed to evaluate the factors that significantly influence the strength prediction model. Scanning electron microscopy (SEM) was used to observe the matrix structure, which was used to evaluate the patterns followed by different UPVs at the same compressive strength. Furthermore, to evaluate the effect of the aggregate and W/C ratio, experiments were conducted with normal-aggregate, light-aggregate, normal-strength, and high-strength concrete materials.

## 2. Materials and Methods

### 2.1. Experimental Plan

The experimental plan followed in this study is presented in Table 2. Previous studies have mentioned the physical properties of coarse aggregates as the most influential factors for UPV; to evaluate the significance of this, the specimens were divided into normal aggregate concrete (NAC) and lightweight aggregate concrete (LAC). LAC was mixed with artificial lightweight aggregate in which coal ash, a by-product of thermal power plants, was artificially extruded and shaped into a round shape. In addition, for quantitative evaluation and prediction in various strength ranges, NAC and LAC specimens with target compressive strengths of 30 MPa (normal strength) and 60 MPa (high strength) were prepared. To evaluate the compressive-strength prediction model for concrete at room temperature, the mechanical properties of concrete at the ages of 1, 3, 7, 28, 56, and 91 days were analyzed. To evaluate the compressive-strength prediction model for concrete based on high-temperature exposure, the target temperatures were set to 100, 200, 300, 500, and 700 °C. The mechanical characteristics of concrete were evaluated in terms of compressive strength and UPV. Subsequently, the F-test, *t*-test, regression analysis, and error test were conducted to statistically analyze the results.

### 2.2. Materials

The physical properties of the raw materials and chemical composition of cement are listed in Table 3. ASTM Type-I ordinary Portland cement of density 3150 kg/m^3^ and fineness 320 m^2^/kg was used. For preparing the NAC, granite-type coarse aggregates of density 2680 kg/m^3^, fineness modulus 7.03, and water absorption ratio 0.68% were used. For preparing the LAC, an artificial lightweight aggregate (coal ash) of density 1470 kg/m^3^, fineness modulus 6.39, and water absorption ratio 8.68% was used. River sand of density 2540 kg/m^3^, fineness modulus 2.54, and water absorption 1.60% was used as the fine aggregate. Polycarboxylic-based super plasticizer was added to improve the workability of high-strength concrete.

### 2.3. Mix Proportion

The mix proportions used in this study are listed in Table 4. The names of the specimens were derived by combining the type of the set concrete (NAC, LAC) and target strengths (30 and 60 MPa). The W/C ratio was set to 0.41 and 0.28 to achieve the target strengths of 30 and 60, respectively. Furthermore, the unit weights of river sand and cement and S/a were set to be the same to focus the evaluation only on the influence of the aggregate type.

### 2.4. Preparation of Specimens

Cylindrical concrete specimens of dimensions 100 × 200 mm were fabricated to evaluate the mechanical properties of concrete based on age and high-temperature exposure [25]. The specimens were cured in water at 20 ± 2 °C for 28 days. They were then cured in a chamber at 20 ± 2 °C and 50 ± 5% humidity until an age of 91 days after mold dislocation. To evaluate compressive strength and UPV, the surface of the specimens was polished using a grinder before mechanical characterization and heating.

### 2.5. Experimental Investigation

#### 2.5.1. Heating Method

**Figure 3:**
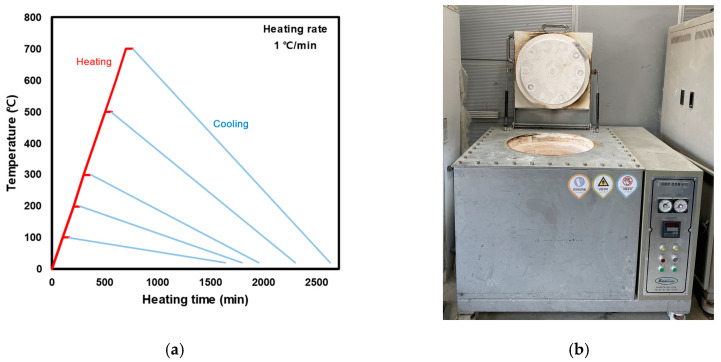
Heating method. (**a**) Heating curve. (**b**) Electric furnace.

Figure 3a shows the heating curve used in this study. After curing the specimen for 91 days, it was heated to the required target temperature (100, 200, 300, 500, and 700 °C) at a low rate (1 °C per min), as suggested by a previous study (Rilem TC 129MHT). Once reached, the target temperature was maintained for 60 min to ensure a uniform temperature distribution. The specimens were heated in an electric furnace, as shown in Figure 3b. The specimens were then cooled in air at a low cooling rate by opening the top cover of the electric furnace until the temperature of the specimens reached 20 ± 2 °C. Subsequently, the mechanical properties of the specimens were evaluated.

#### 2.5.2. Test Method

**Figure 4:**
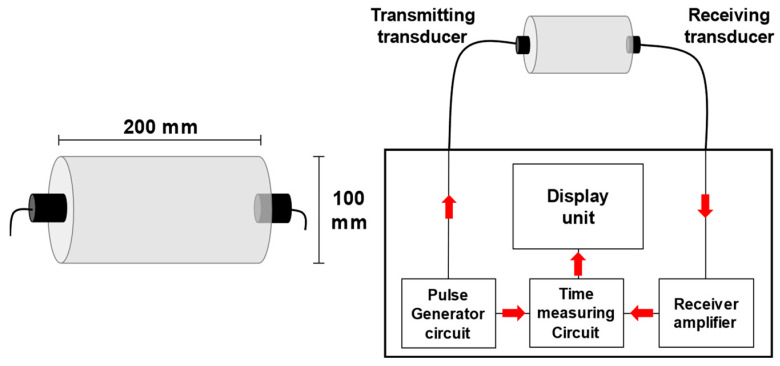
UPV testing.

The test and analysis methods followed in this study are outlined in Table 5. The compressive strength and UPV of the specimens were evaluated following the ASTM C39/C39M and ASTMC597-16 guidelines, respectively [26,27]. Figure 4 shows the front view of the UPV measurement setup. Vacuum grease was applied to ensure a tight contact between the polished specimen surface and ultrasonic probe. UPV data were obtained using an Ultracon-170 Ultrasonic Concrete Tester from MKNDT in Korea with UT transducer of 54 kHz.

To statistically analyze the compressive strength and UPV, each concrete sample was first set and classified into a group according to its type, design compressive strength, and high-temperature history [28]. An F-test was used to evaluate the variance between the mechanical-property data of each group. According to the results of the equal variance test, a *t*-test was conducted under the assumption of equal and unequal variances to evaluate the significant differences between the groups. Subsequently, the compressive-strength prediction model was proposed through regression analysis of compressive strength and UPV data. Finally, the reliability of the model was demonstrated via an error test.

## 3. Results and Discussion

### 3.1. Mechanical Properties of Concrete

#### 3.1.1. Compressive Strength

**Figure 5:**
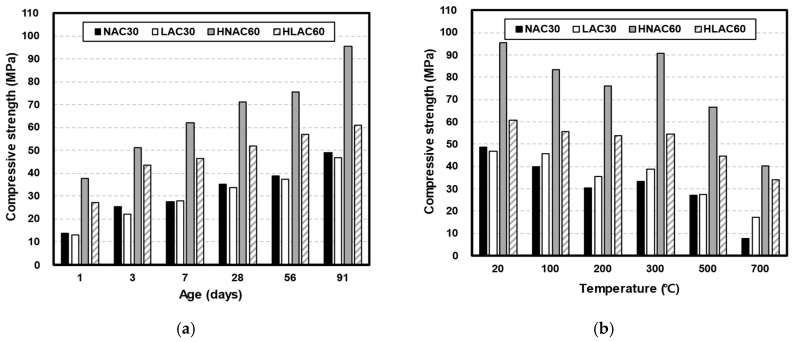
Compressive strength on concrete by age and after high temperature. (**a**) Compressive strength by age. (**b**) Compressive strength after high temperature.

Figure 5 shows the compressive strengths of the specimens with respect to age (Figure 5a) and temperature (Figure 5b). At the same target strength, NAC exhibited a higher compressive strength than LAC for all ages. Previous studies have also reported that concrete blended with conventional lightweight aggregates exhibits low strength development due to the low strength of the aggregates. Vargas et al. analyzed cross-sections of lightweight aggregates from the pumice family via SEM. They reported that cracks develop within the aggregates, presumably during aggregate manufacturing [29]. At an age of 28 days, both NAC30 and LWC30 exhibited a strength of approximately 35.05 MPa and 33.77 MPa, respectively, above the design compressive strengths. However, different results were found at the design compressive strength of 60 MPa. At an age of 28 days, HNAC60 and HLAC60 exhibited a strength of approximately 71.07 MPa and 51.77 MPa, respectively. Vargas et al. and Hossain et al. reported increased stresses in the interfacial transition zone (ITZ) as the cement paste undergoes hydration owing to the ingress of water through the lightweight-aggregate pores during concrete hydration [29,30]. The thickness of the ITZ increased with the increase in W/C ratios. At low W/C ratios, the thickness of ITZ decreased, owing to a more uniform distribution of cement particles near the lightweight aggregate. Hence, the difference between the compressive strengths of NAC and LAC was small under relatively low design compressive strengths; however, this difference increased as the design compressive strength increased. Furthermore, the loading caused only minimal stress because LAC uses lightweight aggregates whose strength is lower than that of hardened mortar. Hence, LAC strength is expected to saturate at high unit volumes of cement [31].

Figure 5b shows the residual compressive strength of the specimens after high-temperature exposure. The temperature-dependent residual compressive strengths of NAC30 and LAC30 exhibited a different trend from their age-dependent compressive strengths. LAC30 exhibited a higher residual strength than NAC30 for all temperature ranges above 100 °C. Concrete is a three-phase composite material, whose properties depend on the influence of the aggregate, paste, and aggregate–paste interface [32].

All concrete specimens used in this study were prepared using the same binder; hence, dehydration-driven degradation of the cement chemical components occurs at the same temperature ranges shown in Table 1. The thermal-expansion properties of the aggregate and the resulting stresses at the interface are also crucial factors influencing concrete properties. When concrete is exposed to high temperatures, cracks form at the interface. This is because of the different thermal-expansion characteristics of the aggregate and paste, which cause the aggregate to expand and the paste to contract. Moreover, lightweight aggregates exhibit a low coefficient of thermal expansion owing to their relatively high porosity; hence, LAC is likely to exhibit a higher residual strength than NAC, owing to the lower stress reduction at the interface [15]. Andiç-Çakır et al. evaluated the high-temperature properties of concrete mixed with limestone aggregate and two types of pumice-based lightweight aggregates. Concrete mixed with limestone aggregate exhibited wide cracks at the aggregate–paste interface at 300 °C, whereas the lightweight concrete exhibited no degradation at the interface except for some cracks inside the aggregate [33]. The residual strength exhibited by LAC30 was approximately 36% higher than that of NAC30. HNAC60 exhibited a higher compressive strength than HLAC60 for all temperature ranges. However, the residual strength ratios exhibited by HANC60 and HLAC60 were approximately 68.20% and 79.75%, respectively. At approximately 700 °C, the difference in the compressive strength between HNAC60 and HLAC60 was minimal, approximately 6.44 MPa.

#### 3.1.2. Ultrasonic Pulse Velocity

**Figure 6:**
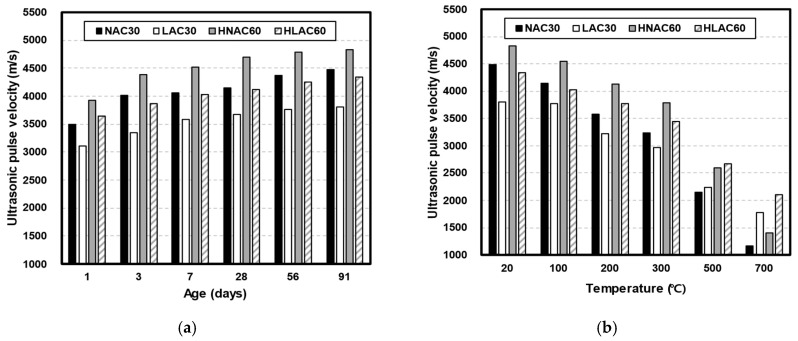
UPV on concrete by age and after high temperature. (**a**) UPV by age. (**b**) UPV after high temperature.

Figure 6 shows the UPV in the concrete samples with respect to age (Figure 6a) and temperature (Figure 6b). For the same target strength, NAC exhibited a higher UPV than LAC, similar to the compressive strength results. The compressive strengths of NAC30 and LAC30 were similar for all ages, and HNAC60 and HLAC60 showed large compressive-strength differences. However, the UPV results showed similar differences at the design compressive strengths of 30 and 60 MPa. As evident from Equation (1), UPV is proportional to the square root of the elastic modulus and is affected by the presence of voids and cracks in the matrix. The lightweight coal-ash aggregate used in this study has a considerably lower elastic modulus than the granite aggregate. Previous studies have also reported that LAC exhibits a lower elastic modulus than NAC [33]. Moreover, the high porosity of lightweight aggregates contributes to the low UPV value. A low W/C ratio increases the UPV in concrete with low porosity and high elasticity, owing to the different hydration products [34]. All mixtures used in this study were prepared using the same binder. The only difference between the mixtures was the type of aggregate used; hence, a different W/C ratio would lead to a different UPV value solely due to the effect of the aggregate. At an age of 28 days, NAC30, HLAC60, and HNAC60 exhibited UPVs of approximately 4147, 4123, and 4696 m/s, respectively. However, LAC30 showed a UPV of approximately 3666 m/s.

Figure 6b shows the residual UPV in the specimens with respect to temperature. LAC showed a higher residual UPV than NAC under temperatures above 500 °C. At 700 °C, NAC30 and HNAC60 exhibited UPVs of approximately 1171 and 1409 m/s, respectively, while LAC30 and HLAC60 exhibited UPVs of approximately 1775 and 2099 m/s, respectively. Furthermore, the UPV retention in LAC30 was approximately 9.76% higher than that in NAC30, similar to their compressive-strength trends. The UPV retention in HLAC60 was approximately 5.62% higher than that in HNAC60. Roufael et al. evaluated the UPV and high-temperature properties of concrete blended with three types of lightweight aggregates: limestone-based aggregates, two types of expanded clay, and expanded shale [35]. The LAC exhibited high UPV retention. Their SEM analysis results revealed that the conventional concrete developed severe cracks at the aggregate–paste interface at 600 °C. However, the LAC did not exhibit any cracks. The researchers hypothesized that this phenomenon may have affected the UPV. Furthermore, the residual-modulus results indicated that LAC has a high residual modulus. Toric et al. also documented a high residual modulus for LAC. This high residual modulus may have contributed to the high UPV retention [36].

#### 3.1.3. Relative Compressive Strength and UPV

**Figure 7:**
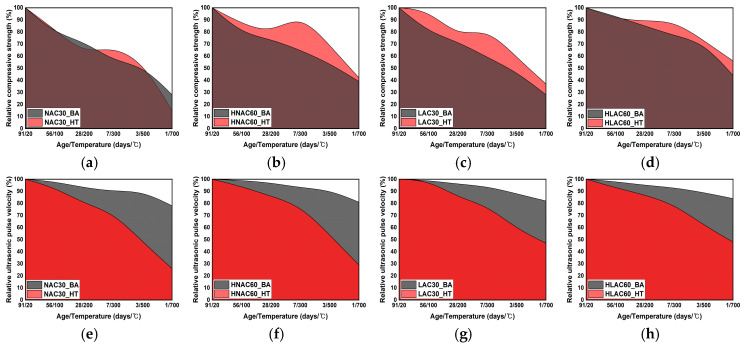
Relative compressive strength and UPV according to room temperature data and high-temperature data. BA (by age); HT (high temperature). (**a**) NAC30. (**b**) HNAC60. (**c**) LAC30. (**d**) HLAC60. (**e**) NAC30. (**f**) HNAC60. (**g**) LAC30. (**h**) HLAC60.

Figure 7 shows the relative compressive strength and UPV at room temperature and high temperatures. Figure 7a–h shows the compressive strength and UPV results, respectively. Except for NAC30, the compressive strength of the specimens after high-temperature exposure tended to be higher than their age-specific compressive strengths. NAC30 exhibited a higher compressive strength after high-temperature exposure than its age-specific compressive strength in the range of approximately 300–500 °C.

Thereafter, the two compressive strengths were similar. HNAC60, LAC30, and HLAC60 showed approximately 20.18%, 24.82%, and 9.53% higher compressive strength after high-temperature exposure compared to their corresponding age-specific compressive strengths. The specimens subjected to high-temperature history were heated after an age of 91 days. After the generation of hydration products and strength development resulting from the hydration reaction had converged, the strength decreased, owing to structural degradations such as interface cracks, matrix collapse, and voids. However, the lower age-specific compressive strength compared to the temperature-dependent residual compressive strength is attributed to insufficient data on the generation of hydration products and strength development during concrete hydration. After high-temperature exposure, the compressive strength of the specimens increased slightly in the range of approximately 200–300 °C, as reported previously for high-temperature concrete properties [37]. Alonso et al. reported an increase in concrete strength in the temperature range of approximately 400–500 °C. They also reported that the rehydration of the new phases, C-S-H and ettringite, at temperatures above approximately 200 °C and the rehydration of nesosilicate (SiO4) and CaO contribute to the formation of C-S-H [38].

The results for UPV showed a different trend than that of the compressive strength. The age-specific UPV tended to be higher than the residual UPV after high-temperature exposure. NAC30 and HNAC60 showed high UPVs of approximately 66.22% and 57.25%, respectively, whereas LAC30 and HLAC60 showed low UPVs of approximately 31.62% and 29.87%, respectively. Zhang et al. investigated the cracks developed and UPV in concrete after exposing the concrete to high temperatures. They reported that the UPV decreased because the ultrasonic waves were delayed owing to their refraction, diversion, and reflection at the cracks and aggregate–paste interface [39]. In particular, they studied the refraction and reflection of the waves at the crack tips and interface for different mineral species. The interior of the concrete shows a relatively intact matrix morphology during its service life, although it may exhibit low elasticity, owing to relatively few hydration products. However, the decrease in the UPV in concrete after high-temperature exposure is attributed to the dominant influence of deteriorations, such as cracks and voids, on UPV.

### 3.2. Statistical Analysis

#### 3.2.1. Statistical Hypothesis Test

Although many researchers have proposed a concrete strength prediction model, the difference between the strength prediction model and its verification for the history of high temperature is insufficient. In this study, a statistical analysis method—hypothesis testing—was used to evaluate significant differences in mechanical properties according to high-temperature history and to evaluate the significance of aggregate density and design compressive strength [40]. The null hypothesis was set that compressive strength and UPV did not show a significant difference according to each set factor (whether high-temperature history, aggregate density, and design compressive strength), and the significance was evaluated through hypothesis testing by setting that significant difference by each set factor as an alternative hypothesis (experimental hypothesis). An F-test was performed to evaluate equal dispersion and heteroscedasticity before assuming each of the set factors as one group and performing a *t*-test for significance evaluation. Through the F-test, it is possible to evaluate the degree of dispersion, that is, the expression range of mechanical properties and the range of degradation due to high temperature. Through the results of the F-test, a *t*-test was performed to evaluate the significance between groups, and it is possible to quantitatively evaluate whether it is a factor that significantly affects the intensity prediction model according to the results of the significance evaluation.

Table 6 lists the mean compressive strengths and UPVs of this study’s specimens. For each specimen, the mean and standard deviation of the mechanical properties (compressive strength and UPV) for the three established factors were evaluated. The mean compressive strengths of both normal-strength and high-strength concrete after high-temperature exposure were 5.05–13.79 MPa higher than their age-specific counterparts. High standard deviations observed in the compressive strength mostly pertained to the high-strength concrete. This variability is attributed to the difference in the strength development and degradation due to the high design compressive strength. In contrast to the compressive strength, the mean age-specific UPVs of both moderate and high-strength concrete were approximately 537–805.9 m/s higher than their temperature-specific counterparts. The standard deviation of UPV was approximately three times higher after high-temperature exposure, which can be attributed to the severe reduction in UPV with concrete degradation.

Table 7 presents the results of the F-test and *t*-test. The significance level is the probability of perceiving the difference as significant when it is not. Typical significance levels used in hypothesis testing are 0.05, 0.01, and 0.001. In this study, the significance level was set as 0.05, as commonly practiced. If the *p*-value is greater than 0.05, the null hypothesis is accepted; if it is smaller than 0.05, the alternative hypothesis is accepted. The F-test results revealed that the age-specific and temperature-specific compressive strengths and UPVs exhibit equal and unequal variance, respectively. Variance measures the deviation of data from the mean and indicates the range of the data. After high-temperature exposure, the value of UPV varied within a wide range compared to the age-specific UPV. This is because cracking severely reduced the UPV. The *t*-test results showed that the age-specific and temperature-specific compressive strengths were not significantly different, except for HNAC60. By contrast, the UPVs showed significant differences with *p*-values below 0.05. The *t*-test results were likely influenced by the low range of UPV after high-temperature exposure. The compressive-strength prediction model for concrete based on age and temperature is likely to exhibit different UPVs at the same compressive strength.

**Figure 8:**
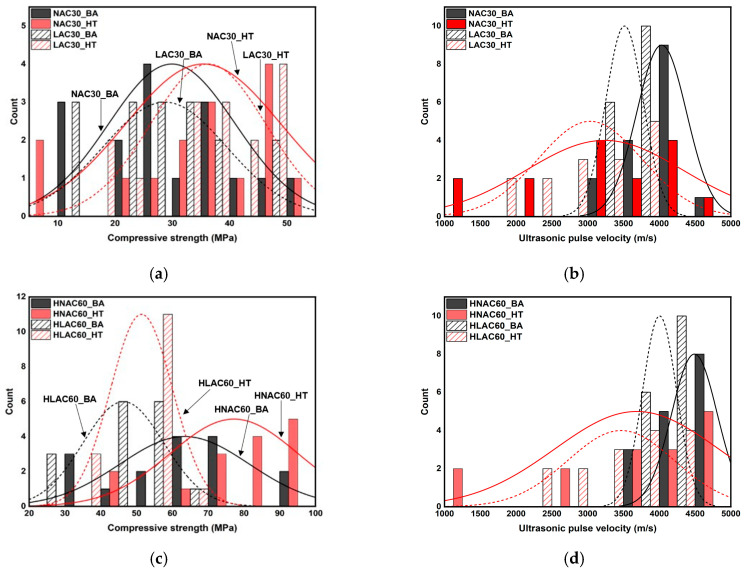
Histogram and normal distribution of mechanical properties on concrete by age and after high temperature. (**a**) NAC30 and LAC30. (**b**) NAC30 and LAC30. (**c**) HNAC60 and HLAC60. (**d**) HNAC60 and HLAC60.

Figure 8 shows the histograms illustrating the distribution of the age-specific and temperature-specific mechanical properties of concrete. After high-temperature exposure, the compressive strengths of NAC30 and LAC30 are primarily concentrated in the range of approximately 35–50 MPa, whereas their age-specific counterparts show a high concentration in the range of approximately 10–35 MPa, with insignificant differences. However, after high-temperature exposure, their UPVs display a wide distribution, and the age-specific UPV is concentrated in the range of approximately 3000–4200 m/s. The two compressive strengths of HNAC60 displayed some significant differences, with a *p*-value of 0.04114, which is less than 0.05 (Table 7). Although significant, this *p*-value is very close to 0.05. This implies that the significance of the difference can be ignored. The UPV in the specimens with a design compressive strength of 30 MPa showed a similar trend, and many data points were above 4500 m/s, owing to the effect of the unit volume of cement.

The F-test results between different aggregate-density groups revealed equal variance, except between the compressive strengths of HNAC60_BA and HLAC60_BA and those of HNAC60_HT and HLAC60_HT. The degree of variance is expected to be different between the compressive strengths of high-strength and normal-strength concretes because LAC exhibits a lower strength than NAC, owing to the limited aggregate strength. The *t*-test results revealed a significant difference between the compressive strengths of HNAC60_BA and HLAC60_BA and those of HNAC60_HT and HLAC60_HT. This is attributed to the effect of the aggregates, as mentioned above. After high-temperature exposure, no significant differences were observed for UPV. Differences in UPV were observed before high-temperature exposure (20 °C); however, the values appeared to converge after high-temperature exposure. This is because of crack formation resulting from the difference in thermal-expansion characteristics of the aggregates and UPV reduction properties.

The F-test results for different design-compressive-strength groups exhibited equal variance, except between NAC30_BA and HNAC60_BA. This is because HNAC60 has a wider range of compressive strengths than NAC30, owing to the high strength expression with respect to age. The *t*-test results showed significant difference, except between the UPVs of NAC30_HT and HNAC60_HT and those of LAC30_HT and HLAC60_HT.

The concrete samples with relatively high design compressive strength before heating (20 °C) showed a high UPV; however, the UPV possibly converged owing to the formation of numerous cracks and voids after high-temperature exposure.

**Figure 9:**
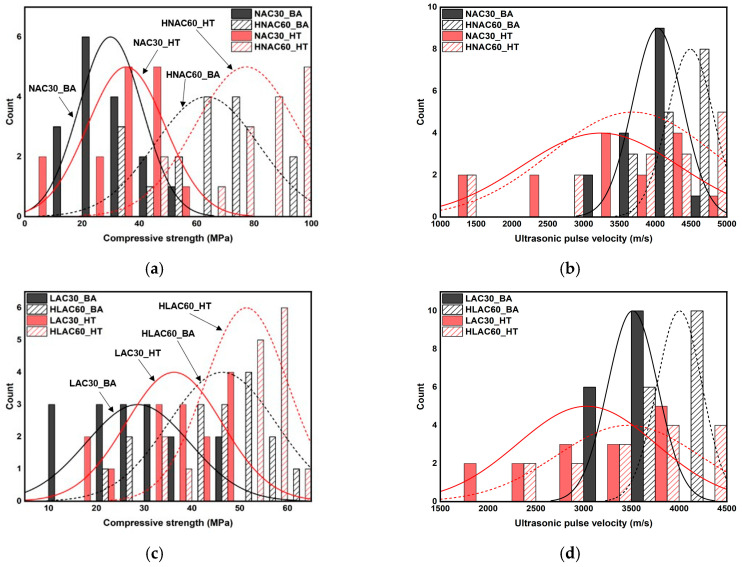
Histogram and normal distribution of mechanical properties on normal-strength concrete and high-strength concrete. (**a**) NAC30 and HNAC60. (**b**) NAC30 and HNAC60. (**c**) LAC30 and HLAC60. (**d**) LAC30 and HLAC60.

Figure 9 shows the histogram illustrating the distribution of mechanical properties of normal-strength and high-strength concrete. It displays a significant difference between the compressive strengths of normal-strength and high-strength concretes. The compressive strengths of NAC30_BA and NAC30_HT were approximately in the range 5–58 MPa, whereas those of HNAC60_BA and HNAC60_HT were approximately in the range 42–100 MPa. LAC also showed a similar trend. The compressive strengths of LAC30_BA and LAC30_HT ranged from approximately 10 to 48 MPa, whereas those of HLAC60_BA and HLAC60_HT ranged from approximately 22 to 65 MPa. The age-specific UPVs showed significant differences; however, they showed a similar distribution after high-temperature exposure.

#### 3.2.2. Regression Analysis

Various forms of compressive-strength prediction models for concrete have been proposed in the literature, including linear, exponential, quadratic, and power functions. The forms were derived by first setting UPV and compressive strength as the independent variable and dependent variable, respectively. Subsequently, regression analysis was conducted, and the regression model was derived [41,42,43,44].

**Figure 10:**
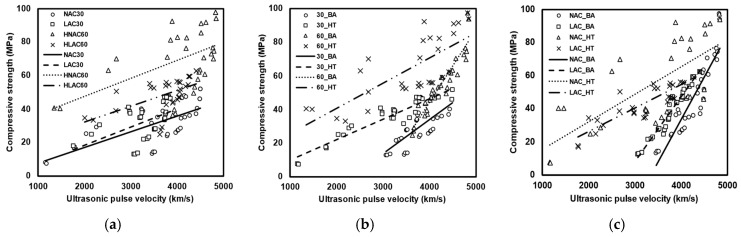
Regression model without consideration of parameters: (**a**) not considering high-temperature exposure; (**b**) not considering density of aggregate; (**c**) not considering design compressive strength.

Figure 10 shows the regression model without considering the parameters. Figure 10a shows the regression analysis results obtained without considering the age-centric and temperature-centric data. All data show a wide range of variance. The specimens with a design compressive strength of 60 MPa show more scatter than those with 30 MPa. Table 8 shows the regression analysis results without parameter consideration. Pearson’s R^2^ (R-squared) is an indicator of the linearity of the data, and R^2^ quantifies the extent to which the independent variables explain the variation in the dependent variable. Without considering the high-temperature exposure of aggregates, Pearson’s R^2^ and R^2^ were approximately in the range 0.56–0.67 and 0.26–0.45, respectively, which are very low values. Figure 10b depicts the regression results without considering aggregate density. 30_BA and 60_HT exhibited relatively low Pearson’s R^2^ and R^2^ values. 30_BA samples exhibited low values due to the difference in UPV, even though the difference between the compressive strengths of NAC30 and LAC30 was non-significant. Moreover, 60_HA samples exhibited low Pearson’s R^2^ and R^2^ values, likely owing to the significant difference between the compressive strengths of HNAC60 and HLAC60.

Figure 10c shows the regression results without considering the design compressive strength. NAC_BA and LAC_BA exhibited high Pearson’s R^2^ and R^2^ values, whereas NAC_HT and LAC_HT exhibited relatively low values. After high-temperature exposure, the residual UPV did not show a significant difference, whereas the residual compressive strength did. The low value may be attributed to the different compressive strengths observed for the same range of UPV. Hence, the design compressive strength must be considered in developing a temperature-based compressive-strength prediction model for concrete.

**Figure 11:**
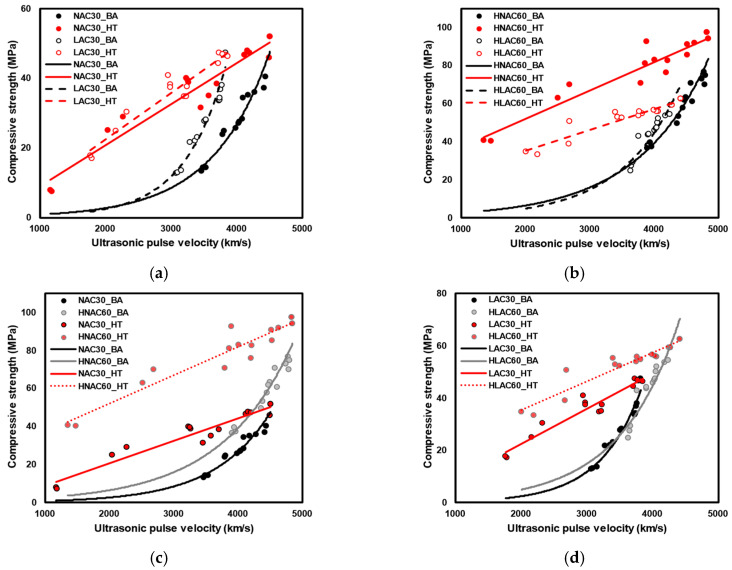
Regression model considering parameters. (**a**) NAC30 and LAC30. (**b**) HNAC60 and HLAC60. (**c**) NAC30 and HNAC60. (**d**) LAC30 and HLAC60.

The regression model produced relatively low Pearson’s R^2^ and R^2^ when the factors set in this study were not considered. The values exhibited by the regression analysis model developed for HA samples were particularly low. Individual analysis of multiple factors may be required for a more accurate evaluation of the structural and material safety of concrete after high-temperature exposure. Figure 11 shows the regression model based on parameter incorporation. The regression model for temperature-specific concrete is a linear function. The regression model for age-specific concrete data is an exponential function. The equations can be expressed as follows:

(2) $$f_{c}=a\times v_{P}+b,$$

(3) $$f_{c}=a\times e^{c\times v_{P}}+b,$$

where $f_{c}$ and $v_{P}$ are the compressive strength (MPa) and UPV (m/s), respectively.

Table 9 lists the coefficients and correlation coefficients of the regression model shown in Figure 11. The compressive-strength prediction model proposed in this study exhibited a high R^2^ value, ranging from approximately 0.87 to 0.94. Pearson’s R^2^ for the temperature-based compressive-strength prediction model was also high, which was approximately in the range 0.94–0.96. The regression model for the age-specific mechanical properties of concrete showed a relatively high R^2^ when the design compressive strength was not considered. However, we believe that design compressive strength needs to be considered for accurate strength prediction.

**Figure 12:**
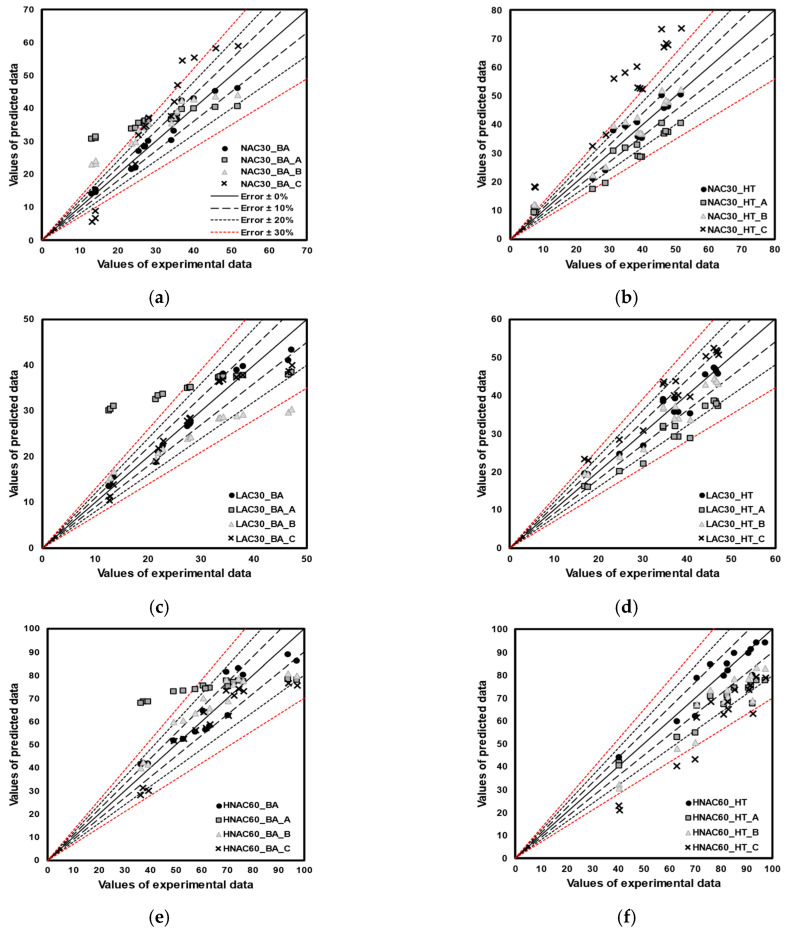

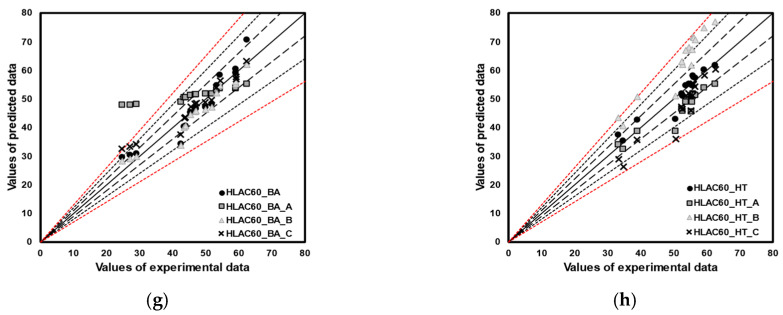
Qualitative error test. (**a**) NAC30_by age. (**b**) NAC30_high temperature. (**c**) LAC30_by age. (**d**) LAC30_high temperature. (**e**) HNAC60_by age. (**f**) HNAC60_high temperature. (**g**) HLAC60_by age. (**h**) HLAC60_high temperature.

Figure 12 shows the qualitative error test results. The reliability of the compressive-strength prediction model with and without considering the factors set in this study was verified through error verification. ‘A’ indicates models developed without considering either age or high-temperature exposure. ‘B’ and ‘C’ indicate models developed without considering the aggregate density and design compressive strength, respectively. Overall, all models displayed similar trends of errors. ‘A’ models based on age displayed a relatively high degree of prediction accuracy for low compressive strengths, whereas temperature-based ‘A’ models exhibited a low degree of prediction accuracy. ‘B’ models developed for NAC30 and LAC30 exhibited similar errors, whereas those developed for HNAC60 and HLAC60 underpredicted and overpredicted, respectively. The ‘C’ developed for LAC and HLAC exhibited errors within 30%; however, NAC and HNAC overpredicted the design compressive strength by 30 MPa and underpredicted by 60 MPa.

**Figure 13:**
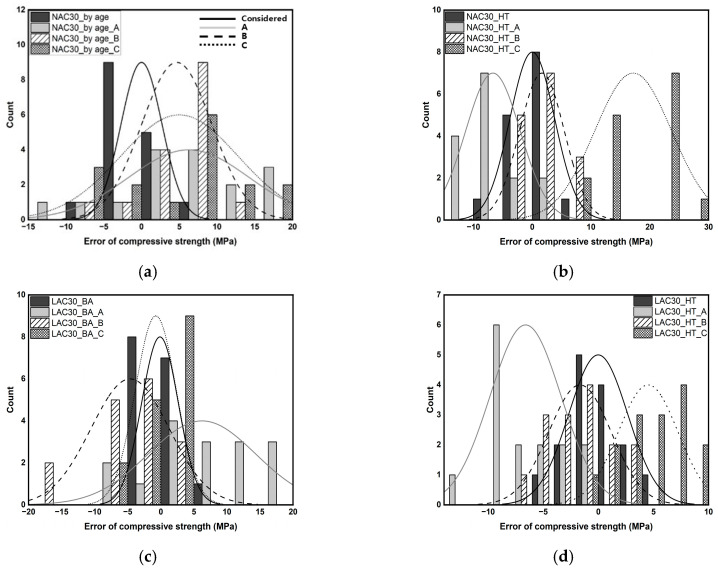

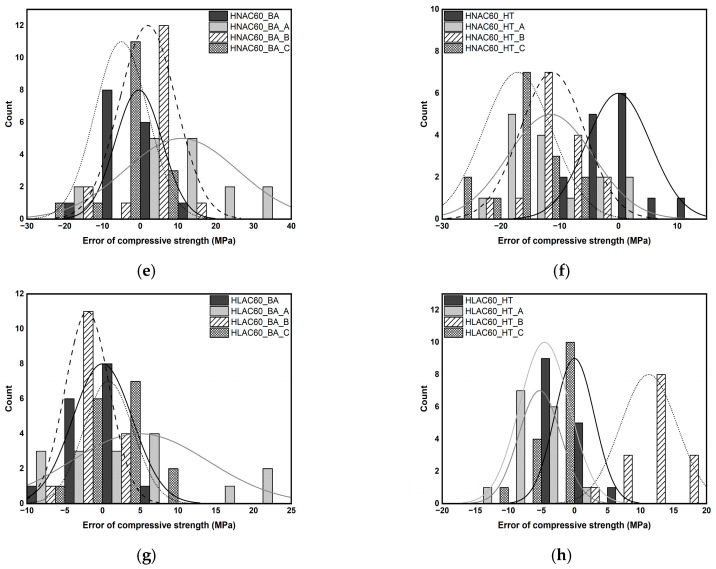
Quantitative error test. (**a**) NAC30_by age. (**b**) NAC30_high temperature. (**c**) LAC30_by age. (**d**) LAC30_high temperature. (**e**) HNAC60_by age. (**f**) HNAC60_high temperature. (**g**) HLAC60_by age. (**h**) HLAC60_high temperature.

Figure 13 shows the quantitative error test results; the mean and standard deviation of the error are outlined in Table 10. The mean error and standard deviation exhibited by ‘A’ models were approximately 7.29 and 7.31, respectively. HNAC60_BA displayed an error range of 30–35 MPa. ‘B’ models exhibited a mean error and standard deviation of approximately 4.89 and 4.68, respectively. HNAC60_HT_B and HLAC60_HT_B displayed an error of approximately ±11.26. An increase in strength range is anticipated to result in a more pronounced influence of aggregates in predicting the strength of concrete after high-temperature exposure. ‘C’ models exhibited a mean error and standard deviation of approximately 5.74 and 5.01, respectively. NAC30_HT_C and HNAC60_HT_C displayed a mean error of approximately ±17.18. The incorporation of design compressive strength is considered crucial for accurately predicting the strength of concrete mixed with ordinary aggregates after high-temperature exposure.

### 3.3. Prediction Model and Microscopic Analysis

**Figure 14:**
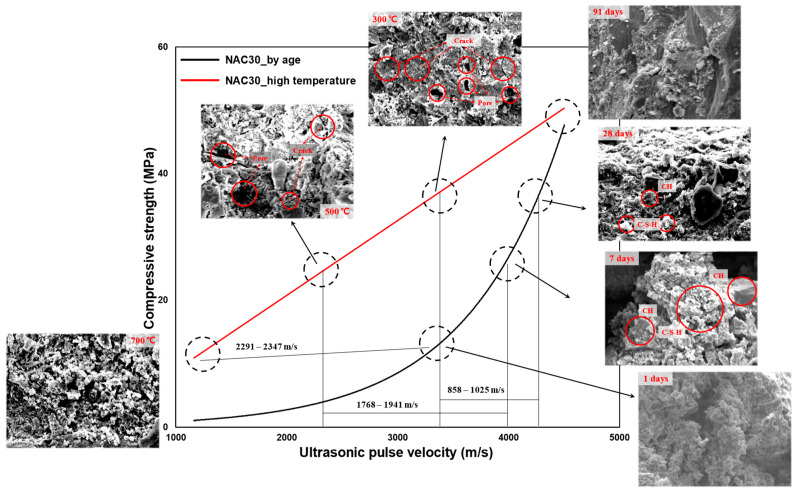
Analysis of regression model differences with SEM results.

Figure 14 shows the results of analyzing the regression model differences, along with SEM images. The existing age-based and temperature-based compressive-strength prediction models for concrete exhibit different results (see Figure 1). In this study, the temperature-based compressive-strength prediction model for concrete showed higher strength than the age-based model for the same UPV range and lower UPV for the same compressive strength. This is attributed to the effect of cracks and voids in the concrete matrix, which were formed after exposure to high temperature, as shown by the compressive-strength prediction model and SEM analysis of the NAC30. At an age of 91 days (before heating), NAC30 displayed a very dense matrix structure. At 300 °C and an age of 28 days, NAC30 exhibited a compressive strength of approximately 35 MPa, and the difference in UPV was approximately in the range 858–1025 m/s. NAC30 did not display severe matrix degradation, although some microcracks and voids were observed at 300 °C [45,46]. At an age of 28 days, NAC30 exhibited the design compressive strength and an intact matrix structure. At 500 °C and an age of 7 days, it displayed a compressive strength of approximately 25 MPa, with more severe differences in UPV in the range 1768–1941 m/s. At 500 °C, the NAC30 matrix developed distinct cracks and voids; a drastically degraded phase was observed in the matrix [47,48]. At an age of 7 days, the structure of the matrix was not as dense as that observed at the age of 28 days; however, large amounts of CH and C-S-H were observed in the matrix. NAC30 displayed similar strengths of approximately 10 MPa at 700 °C and an age of 1 day, with severe differences in UPV in the range of approximately 2291–2347 m/s. At 700 °C, extremely severe matrix degradation was observed. At an age of 1 day, it displayed an immature matrix structure with large amounts of amorphous hydration products and ettringite [48,49]. UPV differs with matrix structures, even for the same compressive strength.

Even with the same compressive strength, different matrix structures depending on the high-temperature history have a great influence on UPV. This study can bring about the economic benefits of securing structural safety and evaluating the exact degree of repair and reinforcement of buildings after a fire, and it is believed that UPV can be used to contribute to engineers and researchers who consider the accurate strength prediction and quality evaluation of concrete.

## 4. Conclusions

This study analyzed age-based and temperature-based compressive-strength prediction models for concrete using UPV. Additionally, this study evaluated the factors that lead to significant differences through statistical tests. The results of the experiments and analysis are summarized as follows:

1. The residual compressive strength of the concrete after high-temperature exposure tended to be in range 9.53–24.82%, higher than the compressive strength of the aged concrete. The higher compressive strength of the concrete after high-temperature exposure compared to the age-specific compressive strength is attributed to insufficient formation of hydration products and strength expression. This is because the formation of cracks and voids reduced the strength, while the formation of hydration products and strength development converged to some extent.
2. The UPV of the aged concrete tended to be 29.87–66.22% higher than the residual UPV displayed after the high-temperature exposure of the concrete. The UPV reduced by the formation of cracks and voids is believed to be more sensitive to the severely degraded matrix structure of the concrete exposed to high temperature.
3. The significance test results suggested that the compressive strength and UPV were significantly different owing to high-temperature exposure, aggregate density, and design compressive strength. In particular, consideration of the set factors is critical for evaluating the mechanical properties of HNAC and HLAC.
4. The regression analysis results showed a very low R^2^ when the age-specific and temperature-specific data of the concrete were not separated. The error test result showed that the average error converged to almost zero when the compressive-strength prediction model considered all the factors set in this study.
5. When SEM analysis was performed based on the same compressive strength in the compressive-strength prediction model for concrete by age and after high temperature, the difference in UPV increased in the lower compressive strength range. This appears to be due to the effect of severe cracks and voids that occur in concrete after high temperature, which resulted in a very low UPV compared to the compressive-strength prediction model for concrete by age.

In this study, different UPVs at the same compressive strength were demonstrated through statistical and SEM analysis results, but additional micro-analysis such as quantitative quantity of hydration products, porosity, and visual analysis of cracks is considered necessary to prove clearer experimental results and hypotheses.

## Figures and Tables

**Table 1:** Dehydration and decomposition of cement after high temperature.

Temperature (°C)	Properties
30–200	Expansion and evaporation for physically bound water
80–130	Dehydration of calcium silicate hydrate (C-S-H)
250–270	Dehydration of ettringite (C3A3CSH32)
50–300	Dehydration of monosulfate (C3ACSH12) Three stages of dehydration (50–150 °C, 200 °C, and 300 °C)
450–550	Decomposition of calcium hydroxide (CaO) Ca(OH)_2_ → CaO + H_2_O
570	Phase change of quartz (aggregate)
600–900	Decomposition of calcium carbonate (CaCO_3_)
1100–1200	Melting of concrete

**Table 2:** Experimental plan.

Item	Details
Types of concrete	NAC (normal-aggregate concrete) LAC (lightweight-aggregate concrete)
Design compressive strength	30 MPa (normal-strength concrete) 60 MPa (high-strength concrete)
Curing age	1, 3, 7, 28, 56, 91 days
Heating temperature	100, 200, 300, 500, 700 °C
Mechanical properties	Compressive strength UPV (ultrasonic pulse velocity)
Statistical analysis	F-test, *t*-test, regression analysis, error test

**Table 3:** Physical properties of materials and chemical composition of cement.

Item		Details
Cement		ASTM Type-I ordinary Portland cement Density: 3150 kg/m^3^; fineness: 320 m^2^/kg
Coarse aggregate		Granite aggregate Density: 2680 kg/m^3^; fineness modulus: 7.03; absorption: 0.68%; Size_max_: 20 mm
		Coal-ash aggregate Density: 1470 kg/m^3^; fineness modulus: 6.39; absorption: 8.68%; Size_max_: 20 mm
Fine aggregate		River sand Density: 2540 kg/m^3^; fineness modulus: 2.54; absorption: 1.60%
Super plasticizer (SP)		Polycarboxylic-based super plasticizer
Chemical composition (%)	CaO	60.30
	SiO_2_	19.80
	Al_2_O_3_	4.90
	Fe_2_O_3_	3.30
	MgO	3.80
	SO_3_	2.90
	K_2_O	1.10
	Others	0.90
	L.O.I.	3.00

**Table 4:** Mix proportions.

Mix ID	Ratio		Unit Weight (kg/m^3^)				
	w/c	S/a	Water	Cement	River Sand	Granite Aggregate	Coal-Ash Aggregate
NAC30	0.41	0.46	165	400	799	938	-
LAC30	0.41	0.46		400	799	-	530
HANC60	0.28	0.43		600	676	896	-
HLAC60	0.28	0.43		600	676	-	507

**Table 5:** Test and analysis method.

Item	Standards and Methods
Compressive Strength	ASTM C39/C39M
UPV	ASTMC597-16
F-test	Equal-variance test of two groups
*t*-test	Compare the means of the two groups
Regression analysis	Determine the effect of an independent variable on a dependent variable
Error test	Comparison of experimental and predicted values

**Table 6:** Results of property analysis between groups.

Classification	ID	Compressive Strength		UPV	
		Mean	Standard Deviation	Mean	Standard Deviation
Normal strength	NAC30_BA	29.93	11.08	4032.88	348.50
	NAC30_HT	35.40	13.56	3236.30	1101.62
	LAC30_BA	28.69	10.83	3514.00	262.65
	LAC30_HT	36.26	10.06	3032.67	724.15
High strength	HNAC60_BA	63.43	18.06	4490.63	320.83
	HNAC60_HT	77.22	17.86	3684.73	1145.63
	HLAC60_BA	46.35	11.28	4004.19	238.14
	HLAC60_HT	51.40	8.72	3466.20	746.92

**Table 7:** Results of F-test and *t*-test.

Classification	Groups	Mechanical Properties	F-Test (*p*-Value)	*t*-Test (*p*-Value)
Significance test of mechanical properties by age and after high temperature	NAC30_BA and NAC30_HT	Compressive strength	0.224	0.227
		UPV	<0.050	<0.050
	LAC30_BA and LAC30_HT	Compressive strength	0.394	0.053
		UPV	<0.050	<0.050
	HNAC60_BA and HNAC60_HT	Compressive strength	0.48516	<0.050
		UPV	<0.050	<0.050
	HLAC60_BA and HLAC60_HT	Compressive strength	0.172	0.176
		UPV	<0.050	<0.050
Significance test of mechanical properties according to aggregate density	NAC30_BA and LAC30_BA	Compressive strength	0.465	0.751
		UPV	0.142	<0.050
	HNAC60_BA and HLAC60_BA	Compressive strength	0.039	<0.050
		UPV	0.130	<0.050
	NAC30_HT and LAC30_HT	Compressive strength	0.138	0.846
		UPV	0.064	0.554
	HNAC60_HT and HLAC60_HT	Compressive strength	<0.050	<0.050
		UPV	0.061	0.541
Significance test of mechanical properties according to the design strength	NAC30_BA and HNAC60_BA	Compressive strength	<0.050	<0.050
		UPV	0.376	<0.050
	LAC30_BA and HLAC60_BA	Compressive strength	0.438	<0.050
		UPV	0.355	<0.050
	NAC30_HT and HNAC60_HT	Compressive strength	0.157	<0.050
		UPV	0.443	0.284
	LAC30_HT and HLAC60_HT	Compressive strength	0.300	<0.050
		UPV	0.455	0.118

**Table 8:** Regression analysis results without consideration of parameters.

Classification	ID	Pearsons’s R^2^	R^2^
Not considering high temperature and room temperature	NAC30	0.671	0.450
	LAC30	0.579	0.335
	HNAC60	0.513	0.263
	HLAC60	0.557	0.310
Not considering density of coarse aggregate	30_BA	0.757	0.573
	30_HT	0.949	0.900
	60_BA	0.937	0.879
	60_HT	0.758	0.574
Not considering the design compressive strength	NAC_BA	0.919	0.844
	LAC_BA	0.973	0.947
	NAC_HT	0.712	0.507
	LAC_HT	0.884	0.782

**Table 9:** Coefficients and correlation coefficients of the regression model in Figure 11.

ID	a	b	c	Pearson’s R^2^	R^2^
NAC30_BA	2.59750	−15.05774	0.00007	-	0.940
NAC30_HT	0.01181	−2.82703	-	0.960	0.921
LAC30_BA	0.09397	0.76061	0.00160	-	0.934
LAC30_HT	0.01340	−4.36475	-	0.964	0.930
HNAC60_BA	0.00024	35.49031	0.00254	-	0.882
HNAC60_HT	0.01484	22.55900	-	0.951	0.906
HLAC60_BA	0.55290	−0.34598	0.00110	-	0.871
HLAC60_HT	0.01096	13.39931	-	0.939	0.881

**Table 10:** Quantitative evaluation of errors with histograms and normal distributions.

ID	Means	Standard Deviation	ID	Means	Standard Deviation
NAC30_BA	−0.001	2.722	NAC30_HT	0.010	3.797
NAC30_BA_A	6.269	7.975	NAC30_HT_A	−6.686	4.642
NAC30_BA_B	4.667	4.653	NAC30_HT_B	1.651	3.809
NAC30_BA_C	5.016	7.777	NAC30_HT_C	17.175	6.543
LAC30_BA	−0.127	2.776	LAC30_HT	−0.012	2.671
LAC30_BA_A	6.223	8.125	LAC30_HT_A	−6.605	3.217
LAC30_BA_B	−4.643	5.918	LAC30_HT_B	−1.666	2.836
LAC30_BA_C	−0.757	3.110	LAC30_HT_C	4.516	2.717
HNAC60_BA	−0.397	6.211	HNAC60_HT	−0.017	5.472
HNAC60_BA_A	10.856	14.962	HNAC60_HT_A	−11.557	7.246
HNAC60_BA_B	1.974	7.539	HNAC60_HT_B	−11.265	5.480
HNAC60_BA_C	−5.004	7.018	HNAC60_HT_C	−17.178	5.854
HLAC60_BA	0.001	3.967	HLAC60_HT	0.010	3.007
HLAC60_BA_A	4.918	9.110	HLAC60_HT_A	−5.243	3.200
HLAC60_BA_B	−1.997	2.926	HLAC60_HT_B	11.262	4.307
HLAC60_BA_C	0.780	3.272	HLAC60_HT_C	−4.518	3.803

## Data Availability

The data presented in this study are available on request to the corresponding author.
